# Prevalence of Potential Indicators of Welfare Status in Young Calves at Meat Processing Premises in New Zealand

**DOI:** 10.3390/ani11082467

**Published:** 2021-08-23

**Authors:** Anna L. Palmer, Ngaio J. Beausoleil, Alana C. Boulton, Naomi Cogger

**Affiliations:** 1 School of Veterinary Science, Massey University, Private Bag 11 222, Palmerston North 4442, New Zealand; a.palmer@massey.ac.nz; 2 Animal Welfare Science and Bioethics Centre, School of Veterinary Science, Massey University, Private Bag 11 222, Palmerston North 4442, New Zealand; n.j.beausoleil@massey.ac.nz (N.J.B.); aboulton4@gmail.com (A.C.B.); 3 EpiCentre, School of Veterinary Science, Massey University, Private Bag 11 222, Palmerston North 4442, New Zealand

**Keywords:** bobby calf, welfare indicators, slaughter, lairage

## Abstract

**Simple Summary:**

In New Zealand, every year, more than two million calves between four and seven days of age are sent to meat processing premises. At present, the only information related to calf welfare that is routinely collected is the mortality rate before slaughter. This study aims to describe the status of calves in lairage using animal-based indicators of welfare status that are non-invasive and easy to evaluate. We made 23 visits to 12 meat processing premises across New Zealand. Data collection involved a group-level assessment of nearly 6000 calves in 102 pens, followed by non-invasive measurements on 504 individual calves. We rarely observed calves coughing or engaging in play behaviour. In each pen, faecal soiling occurred on at least one calf, and the percentage of calves affected in each pen ranged from 1% to 48%. Observation on individual calves found that more than 60% had signs of dehydration, and nearly 40% had some faecal soiling present. In conclusion, the most commonly observed potential indicators (dehydration and diarrhoea) were linked to conditions known to be common causes of morbidity, mortality, and compromised welfare. Both dehydration and diarrhoea can be easily assessed in the field, making them potential indicators of welfare for young calves during transport and lairage.

**Abstract:**

In New Zealand, over two million dairy calves between four and seven days of age are sent to meat processing premises every year. There is a need to develop protocols for holistically assessing the welfare of calves sent to slaughter in the first week of life. This study aimed to determine the prevalence of potential animal-based indicators of the welfare state in bobby calves in lairage. The study observed calves in lairage between June and October 2016. Data collection involved assessing groups of calves in pens followed by non-invasive measurements on up to five calves within each pen. We made 23 visits to 12 meat processing premises with group-level observations made on 5910 calves in 102 pens, followed by a non-invasive examination of 504 calves. During the group level observations, none of the calves had their heads tilted or were panting, and coughing and play behaviour were observed in only 1% of pens. In contrast, at least one calf had faecal soiling in all the observed pens, with the percentage of calves affected in each pen ranging from 1% to 48%. In the individual observations, more than 60% of calves had signs of some degree of dehydration, and nearly 40% had some faecal soiling present. In addition, 24% of calves had a respiratory rate over 36 breaths per minute, considered higher than normal. The change in prevalence of some indicators—as time spent in lairage increased or as the calving season progressed—is worth further exploration. Identification of prevalent animal-based indicators facilitates better understanding of the welfare status of young calves in lairage, and these should be incorporated into more holistic calf welfare assessment schemes.

## 1. Introduction

Every year in New Zealand, over 2 million dairy calves (less than one week old) are transported from the farm where they are born to meat processing premises [1,2,3]. These calves are called “bobby calves” and are either male offspring or females that will not be sold or used as replacements within the herd. In New Zealand, dairy farming, and by extension the bobby calf season, is seasonal. Typically, the bobby calf season is 14 weeks long. There is no consistent start date for the season, as the more southern regions tend to calve later.

Bobby calves are at particular risk of compromised welfare due to the young age at which they are transported and held in lairage prior to slaughter [4,5]. Therefore, the transport and slaughter of bobby calves is tightly regulated [6]. For example, the regulations require calves to be at least four days old, have firm hooves and a dry navel. In addition, the calves must show no signs of ill health, have a bright and alert demeanour, and be strong enough to stand, move freely and walk onto the truck without assistance. Calves must also have been fed within 24 h of slaughter, and animals cannot be kept in lairage for more than 20 h.

Across the season, the mortality associated with the transport and lairage of these young calves is less than 1% [7]. Furthermore, the rate has decreased to less than 0.2% in recent years. However, mortality rates alone are insufficient to monitor welfare. There is a need to develop a framework for evaluating the welfare state of young calves in the period leading up to slaughter. A logical first step in developing a framework to evaluate calf welfare status would be to determine how commonly various animal-based indicators occur in the population. Animal-based indicators describe the animal’s response to the environment or management system [8,9]. Unfortunately, there is currently little information on the frequency with which potential indicators of compromised welfare are observed in young calves sent to slaughter.

This study aims to determine the frequency of potential animal-based indicators of poor welfare status in bobby calves held in lairage at New Zealand meat processing premises. The indicators selected were those that could be measured in the field without equipment. The study is not an attempt to design a system to assess welfare or assess the feasibility of such a system. Instead, the results would inform the development of such a tool. We did not set out to determine factors associated with the occurrence of indicators. Therefore, information about farm management or transport of the calves was not captured. However, a decision was made post-hoc to explore the impact of transport time and weeks since the start of the study on potential welfare indicators. Time since the start of the study and time in lairage were selected because of the seasonal nature of dairy farming in New Zealand. Further, previous studies and research undertaken concurrently with this study have demonstrated that the risk of calf mortality increases as the calving season progresses and the time spent in lairage increases [10,11].

## 2. Materials and Methods

### 2.1. Overview

Bobby calves in lairage were observed at 12 meat processing premises in eight different regions across New Zealand between 28 June and 25 October 2016. A multi-stage random sampling method was used to select pens and individual calves for observations. Firstly, pens of calves in lairage were randomly selected, and group-level data was measured by one researcher (AP). Following the group-level evaluation of the calves in each pen, one researcher (AP) entered the pen and randomly selected five calves for closer inspection. A second researcher (AB) recorded the group and individual measurements on standardised pro forma (Supplementary Materials).

### 2.2. Selection of Premises, Pens, and Animals

Meat processing plants were included in the study if they were: (1) in the region the researchers visited for the case-control study run concurrently with this project [11]; (2) the premises expected a minimum of three consignments of calves on the day researchers planned to visit; and (3) the slaughter schedule would allow for observations on the day of data collection. That is, the timing of data collection would not disrupt the yard staff or veterinarians conducting routine antemortem inspections of calves before slaughter.

The day of the week and time of day, and number of times a single meat processing plant was visited varied. Researchers attempted to arrive to maximise the time available for measurements. However, the reality of conducting research in a fully operational slaughter plant during their busy time of the year is that schedules change quickly. Therefore, researchers needed to decide each visit how many pens they had time to sample and then randomly select pens from those in which all calves in the enclosure had been in lairage for at least an hour. The one-hour minimum in lairage was used to allow calves time to settle after travel and unloading. The time in lairage was defined as the number of minutes that had elapsed between calves arriving at the plant and the time that researchers started to make observations. Researchers took the arrival time from the truck docket or records maintained by the meat processing premise.

For each of the selected pens, the researcher collected group level measurements and recorded the number of calves present, pen area, type of flooring, presence, and cleanliness of the water. After taking group level measurements in each pen, five calves were selected for closer examination. The decision was made to select only five calves per pen because a single pen contains animals from the same truck and often the same farm. That is, calves were clustered within pens. Given the aim was to generate population-level estimates of the prevalence of potential animal indicators of welfare, it was preferable to increase the number of clusters, in this case, pens, rather than sampling more animals from within the same cluster [12].

The researcher, after making group-level observations, entered the enclosure at one corner and crossed the pen diagonally at a constant pace. She then followed the fence line to the next corner and crossed the other way diagonally before following the fence line to the final corner. When a calf was asleep on the walkthrough, the researcher walked around the calf and then returned to the planned route.

During the systematic walkthrough, five calves were chosen randomly, using a random number table. When the researcher entered the enclosure, she would start counting calves, with the scribe notifying the researcher when the target calf was reached. A strip of duct tape was then attached to the calf’s back to identify it for later assessment. The researcher inside the pen would then continue counting calves, and the scribe would again notify the researcher when the next calf was to be marked. The process continued until five calves had been selected.

### 2.3. Collection of Group-Level Data

Researchers determined the total number of calves present in the pen and group level before completing the walkthrough. The researchers started by determining the total number of calves and then the number observed to have or display the potential welfare indicators in Table 1. When calves needed to be observed more closely, binoculars were used. During the walkthrough, researchers observed if any of the calves noted as lying down stood up. Attempts were made to measure some variables on a scale; however, ensuring consistent categorisation was difficult. Therefore, the decision was made to simply note if the indicator was present or absent on each animal when measuring variables at the group level. Results were then reported as the percentage of calves in the group in which the indicator was observed.

### 2.4. Collection of Data from Individual Animals

After the group level observations, researchers inspected the five calves selected during the walk through. Except for play and huddling behaviour, all variables measured at the group level were measured at the individual calf level. The disturbance created by having the researcher in the pen made it challenging to measure play and huddling behaviour at the individual animal level. The aim was to observe the calf for two minutes from approximately one meter away. However, the distance was never measured because the behaviour of the other calves made this impossible. Attempts were made to measure some variables on a scale. However, ensuring consistent categorising was difficult, and a decision was made early in the study to record all variables, except injury, as present or absent. When calves had an injury or wound, a five-point scale developed by Jørgensen and colleagues was used to classify the injury [13]. The five levels were: (1) the injury involved only hair loss; (2) the injury involved moderate swelling with, or without, a superficial wound; (3) the injury was a minor cut through the skin or noticeable swelling; (4) the injury went through skin, and there was underlying damaged, and (5) the injury resulted in a loss of function.

At the end of the two-minute observation period, the researcher measured the respiratory rate and then performed the skin tent test. The calf’s sex and breed were also recorded. For the respiratory rate, the number of breaths was counted for 20 s and converted to breaths per minute. Attempts were made to assess whether breathing was hampered or the animal was panting. However, it was not possible to develop a definition that could be consistently applied. For the skin fold test, calves were left in the posture in which they were observed and were gently positioned with their head up and facing straight ahead. The researcher’s right hand was placed with the lateral edge resting lightly against the calf’s scapula. A firm pinch of skin was taken between the thumb and index finger, cranial to the border of the scapula on the calf’s neck. The fold of pinched skin was immediately released, and a stopwatch was activated at the time of release and stopped when the skin had returned to the flattened position and was no longer moving.

### 2.5. Statistical Analysis

The number of pens with water present and the number with dirty water was determined. The pen area, stocking density and height of the metal grate above a concrete floor were summarised using minimum and maximum.

The group-level indicator data were summarised in two ways. Firstly, for each indicator, the number and percentage of pens with at least one calf exhibiting the indicator were determined. Then, for pens where the indicator was observed in at least one calf, the percentage of calves displaying the indicator was calculated. For those pens in which one or more calves displayed the indicator, the minimum, maximum and quartiles for the percentage of calves displaying the behaviour were reported.

For each of the individual-calf level indicators listed in Table 1, results were presented as the number with indicator present and the percentage of all calves with 95% Confidence Intervals. The percentage and confidence intervals were calculated using the *svydesign* function in the R library survey [14]. The method weighted observations based on the probability of selection and adjusted for pen level clustering.

Histograms were produced to examine the distribution of respiratory rate and return time for the skin tent test. For reporting purposes, respiratory rate was coded as decreased if the rate was less than 24 breaths per minute, normal if between 24 and 36 breaths per minute or increased if the rate exceeded 36 breaths per minute [15]. The number and percentage of calves with increased or reduced respiratory rate were reported. Similarly, the time to return for the skin tent test was reported as the number and percentage of calves with a return time greater than or equal to 2, 3, and 5 s [16]. Again, the percentage and confidence intervals calculation considered the difference in probability of selection and the pen level clustering.

Two-way tables were constructed to separately explore the relationship between nasal discharge and ocular discharge, elevated respiratory rate, and reduced respiratory rate. Two-way tables were also used to examine the relationship between skin tent test times of ≥2, ≥3, and ≥5 s, and the presence of faecal soiling, oral behaviour, elevated respiratory rate, and decreased respiratory rate. The significance of relationships was assessed using the Chi-squared test statistic when the actual and expected cell counts exceeded five; otherwise, the Fisher’s exact test was used.

Separate multivariable models were constructed to determine the effect of time in lairage and weeks since the start of the study on respiratory rate and other indicators, which occurred in more than 20% of calves. The effects of time in lairage and weeks since the study’s beginning on respiratory rate were investigated using a mixed-effects linear regression model. For all other indicators, multivariable logistic regression models were used to determine if there was an association. The method used to build both the logistic and linear regressions was the same. First, a preliminary model was constructed that contained both time in lairage and weeks since the start of the study as fixed effects. The model was then extended to include a two-way interaction term. The interaction term was retained if the likelihood ratio test statistic was significant. The scale of the two predictors against the outcome was assessed through the inclusion of a squared term. The relationship between the explanatory variable and the outcome was considered non-linear if the squared term significantly improved the fit of the model, as determined by the likelihood ratio test statistic. When the relationship was non-linear, the continuous variable was replaced with a categorical variable. When a categorical variable was used, differences between the reference and each level were assessed using the Wald test statistic. Finally, each model was extended to include a random effect accounting for the clustering of calves within pens. The mixed-effects models were constructed using the *MASS* library [17] in R.

R version 3.5.1 [18] was used for all analyses, and the level of statistical significance was *p* < 0.05.

## 3. Results

Over an 18-week study period, 23 visits were made to 12 meat processing premises across eight regions of the North and South Islands of New Zealand: six premises were visited only once, three were visited twice, one was visited three times, and two were visited four times. Group-level observations were made on 5910 calves in 102 pens, and 504 individual calves were examined. The pen area ranged from 8 to 120 m^2^ and median stocking density was 0.44 calves/m^2^ (minimum = 0.21 calves/m^2^; maximum = 2.72 calves/m^2^). Metal grate flooring was found in all the pens, and the grate was raised between 10 and 200 cm above a concrete floor. Water was available in all the pens, although the water was not clean in 21 of the 102 pens. The number of pens and calves by plant and weeks since the start of the study are described in Table 2. Logistics on the day (e.g., plant staff needed researchers to move) meant that less than five animals were observed in three pens. The decision was made to retain the pens and calves in the subsequent analysis as the statistical methods were designed to address differences in the probability a calf was selected.

**Figure 1:**
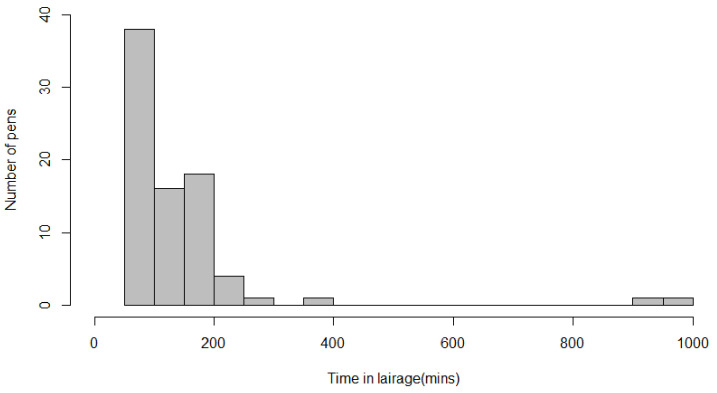
Frequency histogram for the time in lairage before the start of pen walkthrough.

Table 3 displays the percentage of pens for which at least one calf was observed to display the indicator and the percentage of calves in the pen that displayed the indicator. In the observed pens, the median time that calves were in lairage before the walkthrough was 100 min (minimum = 60 min; maximum = 16.5 h; Figure 1).

**Figure 2:**
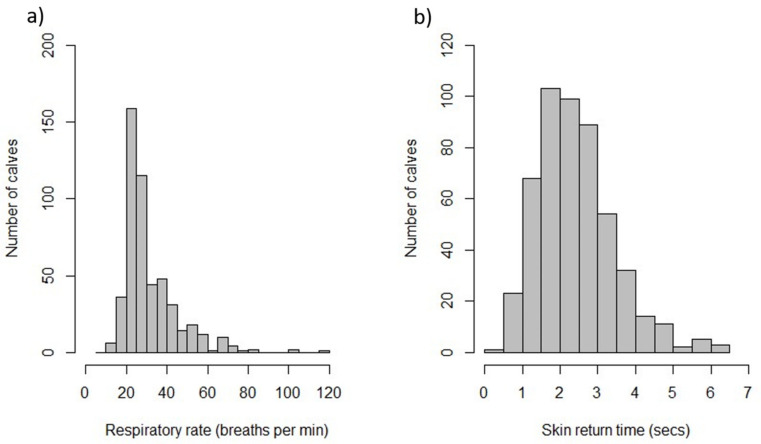
Frequency histogram of respiratory rate (**a**) and the time for the skin to return to normal in a skin fold test (**b**).

For the 504 calves on which individual observations were made, 291 (69%) were male. Breed information was not available for 79 calves. For the 425 in which a breed was recorded: 108 were Friesian, 49 were Jersey, 193 were described as a Jersey Cross, or Kiwi cross, and 75 were Friesian crossed with a beef breed. None of the 504 calves coughed or had a head tilt, although 22% of calves had a respiratory rate over 36 breaths per minute (Table 4). The distribution of respiratory rate and return time in the skin tent test is shown in Figure 2. One hundred and ninety-three calves were lying. Three were in lateral recumbency, and the remainder were in sternal recumbency. For the 22 calves observed to have injuries: eight had hair loss only, eight had moderate swelling or a superficial wound, four calves had a minor cut through the skin or noticeable swelling, and two calves had a wound through the skin with deeper tissue damage. There was no statistically significant association between return time in the skin tent test and the presence of faecal soiling (*p* = 0.21). However, elevated respiratory rate was 1.43 times (95% CI: 1.05–1.96) more likely in calves with nasal discharge than those without (*p* = 0.03).

The model found the respiratory rate decreased by 1.16 (95% CI = 0.54–1.78) breaths per minute for every hour the calf had been in lairage before observation (*p* = 0.006). In contrast, the number of weeks since the start of the study was not significantly associated with the respiratory rate (*p* = 0.08). Mixed-effects logistic regression models found no statistically significant association between weeks since the start of the study and the odds that (1) a skin tent test return was ≥3 s, (2) ocular discharge was present, or (3) that the calf was lying (Table 5). However, as the weeks since the start of the study increased, the odds that the skin tent test return was ≥2 s and that nasal discharge and faecal soiling would be present increased while the odds a calf would display oral behaviour decreased.

The relationship between the time a calf had spent in lairage and skin tent return time, ≥3 and ≥2 s, was non-linear. Therefore, a categorical variable was used for a time in lairage when exploring its effect on skin tent test return times. The categorical variable for the time in lairage significantly improved the fit of both models. When the outcome was that the skin took ≥ 3 s to return to normal in the skin tent test, there was not a significantly different return time between calves that had been in lairage for less than 90 min and those in lairage for 90 to 120 min. In contrast, the odds of skin return time ≥ 3 s were 2.2 times higher in calves that had been in lairage for more than 120 min than for those that had been there less than 90 min.

## 4. Discussion

This study aimed to assess the prevalence of potential animal-based indicators of poor welfare status in bobby calves held in lairage and evaluate the effects of time since the study started and time in lairage on the prevalence of those indicators. Parameters of interest were assessed at the group level across more than 100 pens and individually in more than 500 calves. As expected, more than two-thirds of the calves were male, and most were Friesian or Jersey crossbreeds. When assessed individually at close range the following indicators were observed in 20% or more calves: delayed return times in the skin tent test, faecal soiling, nasal and ocular discharge, and higher- and lower-than-normal respiratory rate. Faecal soiling was the only prevalent physiological indicator when assessed at group level from outside the pen (median prevalence 16%). The utility of each parameter as an indicator of a calf welfare state is discussed below. Prevalent calf behaviours in lairage were lying and oral behaviours. While oral behaviours reflect compromised welfare in young calves, the value of lying for indicating a poor welfare state in this context is not clear. Parameters, such as shivering, coughing, and injuries, and behaviours, such as head shaking, vocalising, and head tilting, were rarely observed in these calves. These indicators are typically intermittent, and the low prevalence could be simply that the two-minute observation period was not long enough. Regardless the parameters may still be useful as indicators of specific welfare problems when they occur.

### 4.1. Delayed Return Times in Skin Tent Test

Sixty-five percent of individually assessed calves displayed skin return times of two or more seconds in the cervical skin tent test, and 26% had return times of three or more seconds. The skin tent test has been validated in calves, of a similar age as those in this study, as a practical non-invasive measure of hydration status in healthy calves with mild to moderate dehydration [19,20]. In these studies, a return time of ≥3 s has been reported to reflect mild to moderate dehydration in young calves [21,22]. Likewise Constable et al. (1998) found a significant association between percentage body water loss and the skin return times, assessed using a pinch and twist method, in calves with experimentally induced diarrhoea [19]. The simple pinch technique used in the current study might be expected to result in shorter return times than a pinch and twist, suggesting return times of 2 or more seconds could reflect moderate dehydration in our study.

When dehydration was defined as a skin tent test time ≥2 s, almost two-thirds of the calves were moderately dehydrated. The welfare implications of mild to moderate dehydration in otherwise healthy animals appear relatively minor. For example, healthy four-day-old calves deprived of feed and water for 24 h were mild to moderately dehydrated (8.4% body water loss on average) but remained bright and alert with good sucking reflexes throughout [21]. In contrast, moderate to severe dehydration is often accompanied by lethargic/weak behaviour and recumbency in calves [22]. In humans, moderate to severe dehydration is often accompanied with unpleasant feelings of thirst, dry mouth, fatigue, weakness, lethargy, and headache [20]. Ten calves (1%) had skin tent return times of 5 s or longer and may have experienced more significant welfare impacts.

In the current study, we found that time in lairage before the observation period influenced the likelihood of detectable dehydration using the skin tent test. A calf that had been in lairage for more than two hours was more than twice as likely to have a return time of ≥3 s than one that had been lairage for 90 min. The greater risk of dehydration may reflect longer times since calves were last fed [23], although time in lairage provides only part of this information. Calves had been in lairage for a median of 100 min before observation (Range 1 to 16.5 h).

In contrast, several previous studies found no biochemical evidence of dehydration (e.g., no increase in plasma osmolality, total protein or packed cell volume) in healthy young calves deprived of feed for up to 13 h [24], 18 h [25] or 30 h with 12 h of transport [26]. Possible explanations for the different findings in the current study are longer total durations of feed deprivation, that the skin tent test as administered over-estimated the level of dehydration [19], or that dehydration was due to factors other than feed deprivation.

Although many calves showed faecal soiling, skin tent return time was not statistically associated with its presence. However, diarrhoea (and subsequent metabolic acidosis) apparently can occur in young calves without clinically significant dehydration [27]. Thus, the cause (or causes) of the increased skin tent times is unclear and should be explored further.

### 4.2. Faecal Soiling

At the group level, at least one calf in every pen was observed to have some degree of faecal soiling. A median of 16% of calves had faecal soiling that was detectable from outside the pen. In contrast, 38% of individual calves, when observed close-up, displayed faecal soiling. Soiling was only noted as present or absent, and the difference between group and individual-level prevalence likely reflects the challenge of detecting mild soiling from a distance.

The cause of the faecal soiling in this study was not determined, but calves this age are particularly susceptible to both infectious diarrhoea and nutritional scouring (osmotic diarrhoea) [20,28,29]. Infectious diarrhoea is more common [30,31], and the risk is reportedly increased by early removal of calves from their dams and feeding of poor quality colostrum [28,29] and by mixing animals from different farm sources [32,33]. Both causes of diarrhoea may result in severe dehydration, and infectious diarrhoea might also be accompanied by feelings of general sickness, abdominal discomfort, and perianal pain [34]. In addition, infectious diarrhoea may also be associated with electrolyte imbalance and metabolic acidosis, which could cause breathlessness [35,36].

The likelihood that faecal soiling would be observed increased as the calving season progressed. It is noteworthy that an earlier study in New Zealand found that the most common reasons for calf mortality and condemnation were diarrhoea or enteritis and that the risk of mortality and condemnation increased as the season progressed [11]. While neither study determined the cause of the diarrhoea, these findings support a hypothesis that the risk of infectious diarrhoea increased over the season or that seasonal changes to other farm management practices increased the risk of nutritional scouring. In support of the former interpretation, failure of passive transfer of maternal antibodies, which would increase the risk of infectious diarrhoea, has also been shown to occur more frequently in the middle compared to early in the New Zealand calving season [29]. Moreover, calves might travel further towards the end of the calving season when fewer premises are operating, contributing to the increased prevalence of mild dehydration (≥ 2 second skin return) and faecal soiling as the season progressed. Further research should evaluate the cause of faecal soiling and factors across the supply chain that influence its occurrence.

### 4.3. Ocular and Nasal Discharge

Ocular and nasal discharge may be indicative of infection, acute irritation or chronic disease [37]. Depending on the cause, the kind and severity of discharge may indicate poor welfare states. For example, nasal discharge and coughing may be signs of respiratory disease or reflect poor air quality and acute irritation [38].

In this study, at least one calf was observed with ocular discharge in nearly 80% of pens and nasal discharge in half, but these clinical signs were rarely detected within those pens (median 3%). In contrast, both ocular and nasal discharge were prevalent in calves observed from one meter away (24% and 37%, respectively). The difference in prevalence of nasal and ocular discharge between group-level and individual measurements likely reflects the distance from which group-level observations were made.

In almost every case, the secretions were serous, indicating that they were probably due to acute irritation rather than infection. Irritation and discharge may have occurred due to dust, exposure to wind on the transport truck, or noxious gases present during transport or in lairage [39]. Calves were more likely to have nasal discharge later in the season, reflecting seasonal changes in on-farm management or environmental factors.

### 4.4. Respiratory Rate

In this study, the normal range of respiratory rates for calves was taken to be 24 to 36 breaths per minute [15]. According to this definition, almost a quarter of individual calves exhibited abnormally high respiratory rates, and a similar percentage had unusually low respiratory rates in lairage. Other normal ranges cited include 20–40 bpm for ‘calves’ and 58 ± 6.2 bpm for calves in the first week of life. Using a broader definition of normal respiration, fewer than 20% of calves had abnormally high or low rates. For example, only 8.3% of calves had a respiratory rate of less than 20 bpm. Nineteen per cent had a rate of 40 breaths or more, but only 4.8% were higher than 60 bpm.

While hyperventilation could be exercise-related, very few calves were observed in active play. Thus, hyperventilation may reflect respiratory illness, fever or increased chemical drive to breathe due to metabolic acidosis [40,41]. As coughing was rarely observed, and nasal discharge was always serous, respiratory illness seems unlikely to be the leading cause. Metabolic acidosis is most often caused by severe diarrhoea in calves but would usually be expected to be accompanied by moderate to severe dehydration, which was rarely observed. Calves exhibiting severe faecal soiling and dehydration, and abnormally high respiratory rate should be treated or slaughtered immediately.

The most reasonable explanation for the mild hyperventilation observed in around 20% of calves is stress due to recent transport, handling or the novel environment [42]. Supporting this explanation is the finding that respiratory rate decreased by around one breath per minute for each hour calves spent in lairage. While we would expect calves to have settled after at least an hour in lairage, the arrival of stock trucks, movement of animals and associated noises may have caused stress until the calves began to habituate.

### 4.5. Lying and Oral Behaviours

When observed undisturbed from outside the pen, a median of 62% of calves were found to be lying. The calves had been in lairage for a median of 100 min by this time. Still, the likelihood of lying/standing was not significantly influenced by time in lairage. When evaluated at the individual level the rate was lower with only about 40% of the observed calves lying. The lower rate of lying when making individual observations was likely due to the fact that calves stood when the observer walked through the pen.

Care must be taken to interpret behaviours in terms of calf welfare status; prevalence alone does not indicate welfare compromise. While a preference to lie has been associated with severe dehydration in neonatal calves [22], most calves in this study were only mildly dehydrated or not at all. Thus, this would not account for the high prevalence of lying observed. Lying in sternal recumbency is normal for calves of this age, and they spend most of their non-feeding time in this posture [43,44]. Thus, lying is unlikely to be a helpful indicator of welfare status in bobby calves in lairage, except to note that many calves were willing and able to rise from lying when the researcher was inside the pen.

In contrast, oral behaviours, such as sucking or licking objects or pen-mates, are considered abnormal and a sign of reduced welfare due to inappropriate or inadequate environmental stimulation or feeding [44,45]. These behaviours could indicate hunger, boredom, or frustration, all of which are negative experiences that impact welfare detrimentally if intense [45].

There was wide variation in the proportion of calves showing oral behaviours when observed from a distance (2–47%; median 12%), but they were often performed during individual observations. Nearly 30% of all calves showed some form of oral behaviour, and 94% of those involved manipulation of objects. In older calves, object-directed oral behaviours reflect a poorly stimulating environment and redirections of age-dependent grazing, ruminating or exploratory behaviour. For example, in pre-weaned calves offered milk and solids, manipulating objects in the environment increased until solid feed intake stimulated rumination [46]. In very young calves, such as those observed in this study, the meaning of object manipulation is less clear, although it is likely to reflect compromised welfare.

About a quarter of calves expressing oral behaviours were observed sucking or licking other calves (cross-sucking). Cross-sucking is considered to reflect the thwarting of the strong motivation of young calves to suck [46]. The amount of milk/replacer fed also influences the expression of cross-sucking, with calves fed less showing more of this behaviour [45,47]. As such, hunger, restricted milk feeding and the inability to suck can increase the expression of cross-sucking [48]. In very young calves in lairage, this behaviour is likely to reflect compromised welfare associated with inadequate feed intake.

In addition, as noted above, time in lairage does not accurately reflect time since the last feed, as we had no information about how long these calves were transported before arriving at the processing premises. Legally, calves must be slaughtered (or fed) within 24 h of their last feed [6]. To meet the regulations, farmers feed the calves before collection, and drivers record the time they collected their first group of calves. Therefore, it would be reasonable to assume calves had been offered food in the past 24 h. However, this does not mean they are not hungry between feeds.

Finally, the prevalence of oral behaviours decreased as the season progressed. The reason for this finding is not apparent but could be due to more frequent slaughtering during the peak of the season so that calves were held in lairage for a shorter time and did not have to be transported as far to get to an operating premise. In any case, the moderate prevalence of oral behaviours in calves in lairage suggests that their welfare is compromised. Oral behaviours could be a useful indicator to explore how welfare can be improved.

### 4.6. Methodological Considerations

Indicators included in welfare assessment protocols such as the one implemented in this study provide only a snapshot of the welfare state at the time of the observations. Many of the potential indicators measured reflect the welfare state of the animals over a more extended period than the observational period [39]. For example, mild to moderate dehydration due to food deprivation or diarrhoea with an infectious or nutritional origin develop over many hours. Therefore, a single skin tent test may not detect the problem. Likewise, the performance of oral behaviours reflects not only experience of recent food deprivation and current environmental stimulation but also the management on the farm (e.g., feeding regime). Thus, what such indicators tell us about the effects of lairage, specifically on calf welfare, must be considered with caution. On the other hand, evaluation of indicators in lairage may be valuable to inform our understanding of calf welfare across the broader supply chain, i.e., from farm management and transport to the point of slaughter.

Research in the year of the study indicated that about half of calf deaths and condemnation happened shortly after arrival at the plant [11]. In theory, making an observation an hour after calves were unloaded may have resulted in underestimating conditions because calves with compromised welfare will already have died or been killed on welfare grounds. However, the degree to which the estimates in the current study are biased is minimal because the incidence of deaths and condemnations in the study year was 0.12%.

## 5. Conclusions 

The most prevalent potential indicators of welfare observed in this study reflected health conditions known to be common causes of morbidity and mortality in young calves in New Zealand: dehydration and diarrhoea. Other prevalent indicators, such as serous nasal and ocular discharge, probably indicate less serious health-related welfare impacts. Oral behaviours expressed in lairage likely indicate longer-term welfare problems in young calves, relating to inappropriate feeding or environmental conditions. Identification of these prevalent animal-based indicators facilitates better understanding of the welfare status of young calves in lairage prior to slaughter than does mortality alone, and they should be incorporated into more holistic welfare assessment schemes. In addition, the prevalence of these indicators means that they will be useful in evaluating the effects of various management factors on some aspects of calf welfare.

The indicators identified in this study can be quickly assessed in focal individuals in the field without the need for specialised equipment to identify calves at risk of current or developing welfare compromise, allowing interventions as necessary. Ideally, these types of indicators should be assessed from close range, as faecal soiling, oral behaviours, and serous discharges were judged to be much less prevalent when observed from outside the pen. Conducting observations at close range would also facilitate assessing the severity of the clinical sign and co-occurrence of other potential indicators to understand the significance of any welfare compromise better. The finding that the prevalence of delayed skin tent test return times, faecal soiling, nasal discharge, and oral behaviour was affected by time spent in lairage or the number of weeks since the calving season started suggests that the effects of farm management and transport on calf health and welfare should be investigated further.

## Figures and Tables

**Table 1:** Definition of potential welfare indicators recorded at the group or individual level. All variables except for injury are reported as present or absent.

Indicator	Defined
Coughing ^a^	The rapid and noisy expulsion of air from the lungs
Faecal Soiling	The presence of faecal material around the anus, hindquarters, or hind legs
Head Shaking	Repeated rapid movement of the head either side to side, up and down or a combination of both
Head Tilting	Head tilted to one side
Huddling ^b^	Calf standing or lying with at least 50% of its body in contact with another animal.
Injury	Presence of hairless patches, swellings, or lesions
Lying	Calf is recumbent
Nasal Discharge	Discharge from the nose
Ocular Discharge	Discharge from the eye
Oral Behaviours	The expression of non-nutritive oral activities when undisturbed
Play Behaviours ^b^	Calf moving or engaged in social play (e.g., running, kicking, butting, and/or mock fighting)
Shivering	Slow and irregular vibration of the body or parts of the body observed when the calf was undisturbed
Vocalisation	Utterance of sound

^a^ Only analysed at the individual calf level; ^b^ Only the pen level observations were reported.

**Table 2:** Number of pens with bobby calves ^a^ available for selection, the number selected, and the number of calves observed at the group and individual level by plant and day and month in 2016.

Plant ID	Date	Number of Pens		Total Number of Calves	
		Available	Sampled	Group	Individual
1	28 June	8	2	108	10
	8 August	12	6	299	30
	19 September	11	6	500	30
	19 September	9	6	359	30
2	5 July	6	6	254	29
	6 July	8	2	119	10
	15 August	6	6	298	30
3	12 July	3	3	213	15
	13 July	2	2	185	10
4	22 July	3	1	150	3
5	25 July	6	1	26	2
6	28 July	10	2	52	10
	29 July	15	3	72	15
	5 September	14	4	133	20
	6 September	8	3	79	15
7	1 August	9	4	257	20
	12 September	7	6	324	30
8	22 August	8	7	507	35
	5 October	10	7	412	35
9	29 August	15	6	403	30
10	10 October	10	6	513	30
11	19 October	7	6	235	30
12	25 October	7	7	412	35
**Total**		**194**	**102**	**5910**	**504**

^a^ While the number of pens is fixed, not all pens in a slaughter plant would be used for bobby calves.

**Table 3:** Number and percentage of pens in which one or more calf displayed an indicator and minimum, maximum and quartiles for the percentage of calves in the pen exhibiting behaviours or indicators of poor health in pens in which the behaviour was observed.

Welfare Indicator	Number (%) of 102 Pens	Min	Percentile			Max
			25th	50th	75th	
Faecal Soiling	102 (100%)	1	10	16	24	48
Calf did not stand when approached	102 (100%)	2	40	54	72	100
Lying prior to entry	101 (99%)	2	37	62	78	100
Oral Behaviours	100 (98%)	2	7	12	18	47
Vocalizing	87 (85%)	1	3	6	9	27
Huddling	82 (80%)	3	12	21	35	70
Ocular Discharge	79 (77%)	1	2	3	4	14
Nasal Discharge	54 (53%)	1	2	3	4	7
Shivering	26 (25%)	1	2	4	6	17
Head Shaking	37 (36%)	1	1	2	3	8
Play Behaviours	7 (7%)	1	2	2	4	4
Coughing	1 (1%)	-	-	-	-	-
Head Titling	1 (1%)	-	-	-	-	-

**Table 4:** Number and percentage of individual calves exhibiting behaviours and observed to have a potential indicator of poor health.

Welfare Indicator	Number	% (95% CI)
Skin tent test ≥ 2 s	318	65 (57–72)
Faecal soiling	221	38 (32–44)
Nasal discharge	205	37 (30–45)
Lying	193	39 (31–46)
Oral behaviours	139	29 (24–35)
Skin tent test ≥ 3 s	125	26 (21–31)
Respiratory rate ≥ 36	119	22 (16–29)
Ocular discharge	116	24 (18–29)
Respiratory rate ≤ 25	110	25 (18–32)
Injury	22	4 (2–7)
Head shaking	13	2 (1–4)
Skin tent test ≥ 5 s	10	1 (0–2)
Shivering	9	2 (0–5)
Vocalizing	6	1 (0–2)
Coughing	0	0
Head tilting	0	0

**Table 5:** Results of separate mixed-effects multivariable logistics models exploring the effect of weeks since the start of the study and time in lairage on potential indicators of welfare status.

Variable	Beta (SE) ^a^	OR (95% CI)	*p*-Value ^b^
**Skin Return Time in Skin Tent Test ≥ 3 s**			
Weeks since study start	0.005 (0.03)	1 (0.96–1.06)	0.85
Lairage			**0.004**
<90 min	0	REF	
90 to 120 min	0.09 (0.27)	1.1 (0.65–1.87)	0.73
>120 min	0.8 (0.26)	2.22 (1.34–3.69)	0.002
**Skin Return Time in Skin Tent Test ≥ 2 s**			
Weeks since study start	0.06 (0.03)	1.06 (1.01–1.12)	0.03
Lairage			**0.004**
<90 min	0	REF	
90 to 120 min	0.51 (0.33)	1.66 (0.87–3.15)	0.12
>120 min	−0.48 (0.27)	0.62 (0.37–1.05)	0.07
**Faecal Soiling**			
Weeks since study start	0.11 (0.03)	1.11 (1.05–1.17)	0.0002
Lairage	0.04 (0.05)	1.04 (0.94–1.16)	0.45
**Nasal Discharge**			
Weeks since study start	0.23 (0.03)	1.25 (1.18–1.33)	<0.0001
Lairage	0.03 (0.07)	1.03 (0.9–1.17)	0.69
**Ocular Discharge**			
Weeks since study start	–0.01 (0.03)	0.99 (0.93–1.04)	0.61
Lairage	–0.06 (0.06)	0.94 (0.84–1.07)	0.35
**Lying**			
Weeks since study start	0.0001 (0.04)	1 (0.93–1.07)	1
Lairage	0.09 (0.08)	1.1 (0.95–1.28)	0.22
**Oral Behaviour**			
Weeks since study start	–0.08 (0.03)	0.93 (0.87–0.98)	0.01
Lairage	0.04 (0.06)	1.04 (0.93–1.16)	0.5

^a^ SE = Standard Error; ^b^ bold and left-justified *p*-value is for the log-likelihood ratio test statistic. The non-bold right justifies *p*-values are for the Wald test statistic and is for an individual co-efficient.

## Data Availability

Contact the corresponding author.

## Supplementary Materials

The questionnaires are available online at https://www.mdpi.com/article/10.3390/ani11082467/s1.
