# Supplementary material for: Prevalence of Potential Indicators of Welfare Status in Young Calves at Meat Processing Premises in New Zealand
Source: Animals (Basel). 2021 Aug 23;11(8):2467. doi: 10.3390/ani11082467 (PMC8388627; doi:10.3390/ani11082467)
Supplement: Supplementary file 1 [file animals-11-02467-s001.zip › SupplementaryFileA.pdf]

Supplementary file A: Recording form for group level observations used during field observations made at 12 meat processing plants across New Zealand during the 2016 bobby calf season.

Group Level Observations

Pen Number [/]

Time of Arrival [ ] **Time of observations** [ ]

Pen location [ ]

Dimension of pens Length [ ] x Width [ ]

Shelter provided Roof Walls

Temperature [ ]

Wind speed [ ]

Number of animals in pen [ ]

Number of animals lying in the pen [ ] **Standing** [ ]

Behavioural Observations

	Number of animals per group	% of group
**Shivering**		
**Panting**		
**Huddling**		

**Oral Behaviours**		
**Vocalisation**		
**Head tilting**		
**Head shaking**		

Behaviours demonstrating

Health Observations

	Number of animals per group	% of group
**Coughing**		
**Hampered respiration**		
**Severe Ocular discharge**		
**Severe Nasal discharge**		

Faecal Soiling

	Number of animals per group	% of group
No		
Moderately Dirty		
Extremely Dirty		

Injury	# of animals	% of group	
0			No visual wounds/injuries
1			Hair loss
2			Moderate swelling and/or superficial wound
3			Minor cut through skin or obvious swelling
4			Wound through skin with deeper damage
5			Injury resulting in loss of function

Walk-through completed | Y / N | (only if there are calves lying down)

Number of calves lying immediately prior to walk-through ☐

Number of calves that got up when observer first entered the pen ☐

Number of calves that get up during systematic walk ☐

Number of calves that get up when approached within 0.5m (after walk-through) ☐

Number of calves that did not get up (non-ambulatory) ☐

Exhaustion? ☐

Number of calves that return to lying 5minutes after walk-through ☐

Comment on position lying

Water source working

Y / N

Comments and Notes

Diarrhoea
