# Supplementary material for: Prevalence of Potential Indicators of Welfare Status in Young Calves at Meat Processing Premises in New Zealand
Source: Animals (Basel). 2021 Aug 23;11(8):2467. doi: 10.3390/ani11082467 (PMC8388627; doi:10.3390/ani11082467)
Supplement: Supplementary file 1 [file animals-11-02467-s001.zip › SupplementaryFileB.pdf]

Supplementary file B: Recording form for group level observations used during field observations made at 12 meat processing plants across New Zealand during the 2016 bobby calf season.

Individual Level Observations

Pen Number	/	Animal Number	/
Calf standing	Y / N	If no, lying position	

Indicators

Shivering

Yes	
No	

Panting

Normal respiration		20-40 breaths per minute
Mild Heat Stress		Closed mouth breathing w/ respiration elevated over 20-40 breaths/min
Panting		Open mouth breathing with respiration elevated over 40 breaths/min

**Respiration Rate**		breaths per minute
**Hampered respiration**		
**Coughing**		

**Vocalisation**	Vocalising?	Y / N		
	If yes	Rate over 2 minutes		
		Duratio n		

**Oral Behaviours**	Y / N	Manipulating an object		biting, suckling, licking
		Manipulating a calf		cross-suckling
		Tongue Playing		
		Other		

**Head tilting**	
**Head shaking**	

Faecal Soiling

No	
Moderately Dirty	
Extremely Dirty	

Injury

0		No visual wounds/injuries
1		Hair loss

2		Moderate swelling and/or superficial wound where skin not perforated
3		Minor cut through skin or obvious swelling
4		Wound through skin with damage to deeper tissues
5		Injury resulting in loss of function

Ocular discharge present Y / N

If yes: Mild ☐ Severe ☐

Nasal discharge present Y / N

If yes: Mild ☐ Severe ☐

Dehydration Time for skin to return to normal after skin tent:

**General demeanour**	Bright	Tired	Weak
**Navel**	Wet	Raw	Dry
**Eyes**	Bright	Dull	Closed
**Ears**	Forwards	Back	Alert

**Coat**	Dry	Clean	Wet	Dirty
**Hooves**	Strong	Soft/undeveloped		

Sex

Breed

Comments and Notes

Diarrhoea

Lesions

Lameness

Ectoparasitism

Mucous Membranes
